# The invasive butterbur contaminates stream and seepage water in groundwater wells with toxic pyrrolizidine alkaloids

**DOI:** 10.1038/s41598-020-76586-1

**Published:** 2020-11-13

**Authors:** Vaidotas Kisielius, Jawameer R. Hama, Natasa Skrbic, Hans Christian Bruun Hansen, Bjarne W. Strobel, Lars Holm Rasmussen

**Affiliations:** 1 Department of Technology, University College Copenhagen, Sigurdsgade 26, 2200 Copenhagen, Denmark; 2 Department of Plant and Environmental Sciences, University of Copenhagen, Thorvaldsensvej 40, 1871 Frederiksberg, Denmark; 3 Greater Copenhagen Utility HOFOR, Ørestads Blvd. 35, 2300 Copenhagen, Denmark

**Keywords:** Freshwater ecology, Invasive species, Environmental sciences

## Abstract

Pyrrolizidine alkaloids (PAs) are persistent mutagenic and carcinogenic compounds produced by many common plant species. Health authorities recommend minimising human exposure via food and medicinal products to ensure consumer health and safety. However, there is little awareness that PAs can contaminate water resources. Therefore, no regulations exist to limit PAs in drinking water. This study measured a PA base concentration of ~ 70 ng/L in stream water adjacent to an invasive PA-producing plant *Petasites hybridus (Asteraceae)*. After intense rain the PA concentration increased tenfold. In addition, PAs measured up to 230 ng/L in seepage water from groundwater wells. The dominant PAs in both water types corresponded to the most abundant PAs in the plants (senkirkine, senecionine, senecionine N-oxide). The study presents the first discovery of persistent plant toxins in well water and their associated risks. In addition, it for the first time reports monocrotaline and monocrotaline N-oxide in *Petasites* sp.

A popular fairytale *The Ugly Duckling* written in Danish by Hans Christian Andersen in 1843 narrates that the ducklings were born under the leaves of a butterbur: the plant such tall that small children could stand upright under the largest ones^1^. The customized name of this regional plant was not translated into foreign languages. However, the English name butterbur also has a local customary background. It is supposed to have originated from the large leaves that were used to wrap butter during hot weather^2^. Invasive plants, such as the butterbur, and their adverse roles are little recognized by society, especially when they are inseparable parts of the culture or folklore.

Common butterbur (*Petasites hybridus* (G. Gaertn., B. Mey. & Scherb.)) is a flowering plant found in damp nutrient-rich soil and around the shores of fresh water bodies in northern Europe and parts of North America^3,4^. With the aid of powerful rhizomes it can form large populations; its leaves reach up to 70 cm in width on 1 m stems that shade other vegetation and thereby cause soil erosion^5^. In these regions the species is regarded as invasive since it is originally native to southern Europe and western Asia and was introduced to northern Europe around 1350 as a medicinal plant by monks^5–7^. The butterbur expanded from cultivation sites in monasteries, castles and estates to natural habitats across northern and western Europe. Nowadays, it is also marketed and deliberately planted for ornamental purposes, e.g. around local community fire safety ponds or in garden lakes as portrayed in H.C. Andersen’s *The Ugly Duckling*.

The bioactive sesquiterpene constituents in butterbur (petasin, isopetasin) express antispasmodic properties and offer a variety of medicinal applications where the relaxation of muscle and vascular spasms is desired^4,8–10^. Historically, the plant’s leaves and rhizomes were also used to treat or prevent plague, rashes, arthritis, kidney and bladder stones. Its most documented evidence-based modern therapeutic application is the treatment of migraine headaches and allergic rhinitis^4,5,9–12^. However, commercialization of unrefined butterbur extracts is prohibited due to numerous cases of intensive liver damage caused by high concentrations of unsaturated pyrrolizidine alkaloids^4,5,11,13,14^.

Alkaloids are a large class of naturally occurring diverse organic compounds structurally determined by a heterocyclic ring structure containing at least one nitrogen atom. They are produced as secondary metabolites primarily by fungi and plants, also by bacteria and animals^15–20^. Many alkaloids are highly toxic and some contain properties desired for therapeutic or recreational purposes^21^. Atropine and coniine rich extracts have been used for homicide since ancient Rome. Strychnine and anabasine were used as insecticides before the development of a wide range of synthetic pesticides that are comparatively less toxic to humans^22^. For centuries alkaloids have been used as pharmaceuticals (morphine, codeine) and stimulants (cocaine, nicotine)^16,23^. Health authorities regulate specific alkaloid groups to ensure safety of food, tea, medicine, honey, supplements and other products^17,24–26^.

Heterocyclic rings and ample hydrogen bonds determine alkaloid stability and contribute to their high water solubility. The polarity and persistence of alkaloids make them compatible with persistent and mobile organic compounds (PMOCs). PMOCs are emerging contaminants that are rarely detected in the environment due to the lack of available analytical techniques^27^. PMOCs include surfactants, polar industrial chemicals, pharmaceuticals and personal care products^28,29^. Alkaloids applied as pharmaceuticals or stimulants are also commonly found in waste water and sewage sludge, whereas groundwater surveys reveal alkaloid caffeine among the most frequently encountered compounds^23,30–34^.

Pyrrolizidine alkaloids (PA) comprise several hundred compounds based on the heterocyclic two-ring structure necine^16,35,36^ (Fig. 1). PAs are among the most common natural toxins produced by plants as defence chemicals against insects and herbivores^37–40^. The PA composition in PA-producing plants is not necessarily constant and can vary due to climatic and environmental conditions, the age and the part of the plant, as well as discrete genotypes and chemotypes^25,41,42^. The mode of PA toxicity depends on the molecular structure. When ingested, PAs with an unsaturated bond in 1,2 position of the necine unit (Fig. 1) (hereafter called unsaturated PAs) metabolically oxidize and react with proteins and nucleic acids^43–46^ resulting in hepatotoxic, mutagenic and carcinogenic effects^16,37,41,46–50^.

**Figure 1:**
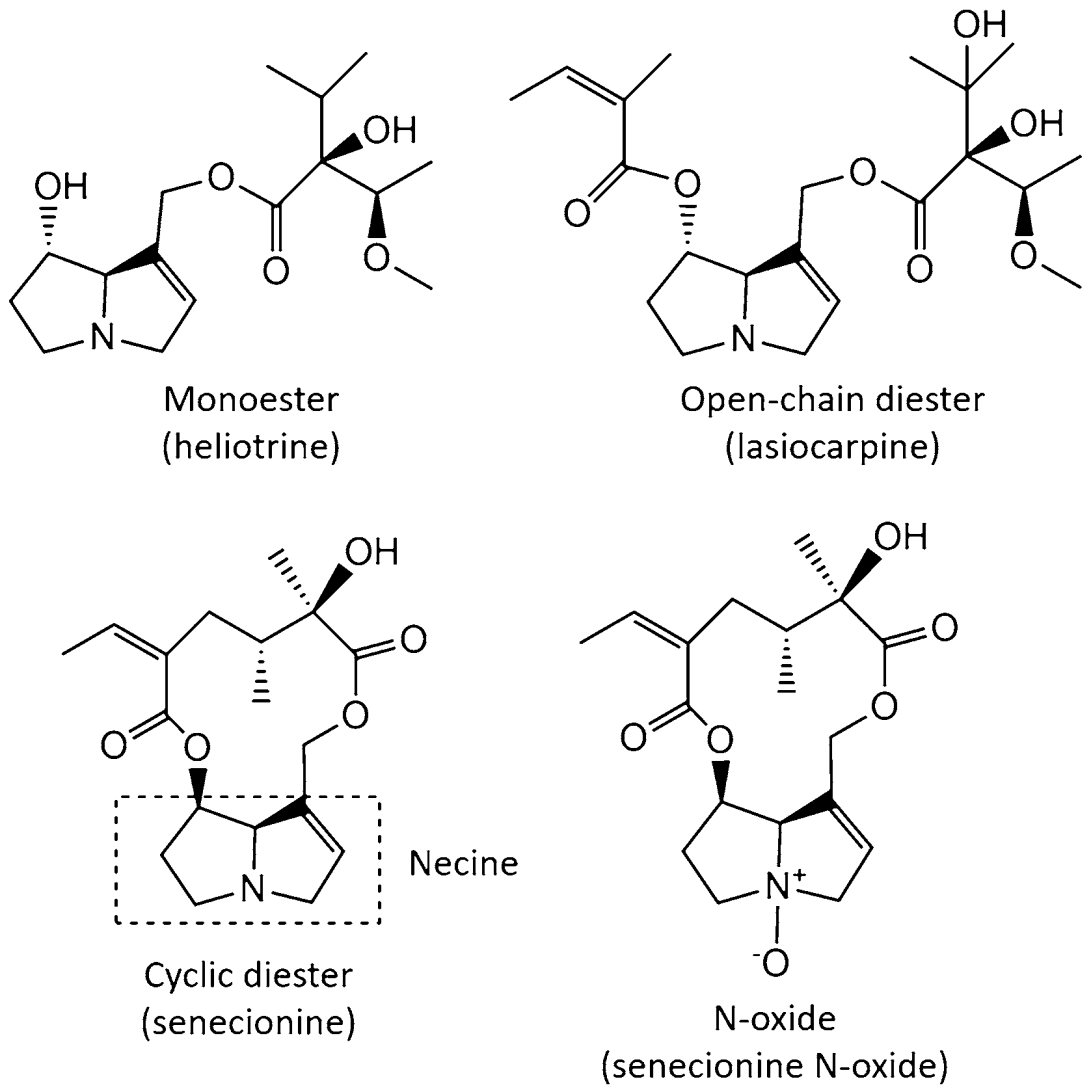
Main structural forms of pyrrolizidine alkaloids (monoester, open-chain diester and cyclic diester) and example of PA metabolite (N-oxide) reported in this study. The unsaturated bond in 1,2 position of the necine is a structural prerequisite for the carcinogenic properties of the compounds.

Oxidized PAs (PA N-oxides, Fig. 1) are present in plants in nearly equal quantities as free base PAs^51^. The toxicity of PA N-oxides has been demonstrated but most are suggested to be less toxic than the PAs from which they originate^42,51,52^. However, metabolic reduction of N-oxides to their corresponding free base PAs has been reported to take place in the gut of animals^42,53^. Studies on human liver microsomal system confirm that the results from animal studies are also relevant to humans^18,51,54^. N-oxides are more polar than free base PAs, implying potentially greater aquatic emissions from plants and a higher mobility in soils and sediments.

Plants with high concentrations of unsaturated PAs are responsible for the death of cattle^18,55^. Toxic effects in humans such as hepatic sinusoidal obstruction syndrome and incidents of acute and subacute food poisoning with high morbidity and mortality have been reported from many countries^24,25,37,38,44,45,50,55,56^. The World Health Organization (WHO) recommends minimizing PA exposure to humans as much as possible^57^. Due to the high toxicity, national health authorities in e.g. Germany and United Kingdom recommended a daily total PA intake of not more than 7 ng/kg bodyweight applying a margin of exposure (MOE) of 10,000^37,58–61^. The European Food Safety Authority (EFSA) recommends minimizing exposure and in 2017 established a benchmark dose lower confidence limit 10% (BMDL_10_) of 237,000 ng/kg body weight per day. Applying the same MOE as above, this results in a recommended threshold of 23.7 ng/kg body weight per day^24,62–64^. The daily limit of 23.7 ng/kg body weight will be used in this study as a reference point.

It is estimated that 3% of all flowering plants contain at least one unsaturated PA^65^. In regions where these plants are prevalent there is an emerging concern of PAs highly exceeding safety limits in honey^20,24,66–69^. Despite their abundance in flora, the degree of natural PA emissions and their fate in the environment remains largely unknown. A recent study measured up to 3800 μg/kg of PAs in topsoil and up to 530 μg/L in pond water. The study associated the compounds with adjacent densely growing common ragwort (*Jacobaea vulgaris* (Gaertn.))^70^.

We hypothesize that PAs can naturally leach from butterbur into water and make it unsafe to drink. The stability of PAs and frequently reported cases of groundwater contamination by structurally similar caffeine imply that PAs can leach into groundwater. Since there are no regulations that require PA monitoring, these and other toxic alkaloids may be overlooked as emerging environmental contaminants. In order to test the hypothesis we measured the content of 21 PAs in a stream, seepage and ground water over a period of four months in a butterbur affected catchment system.

## Results

**Figure 2:**
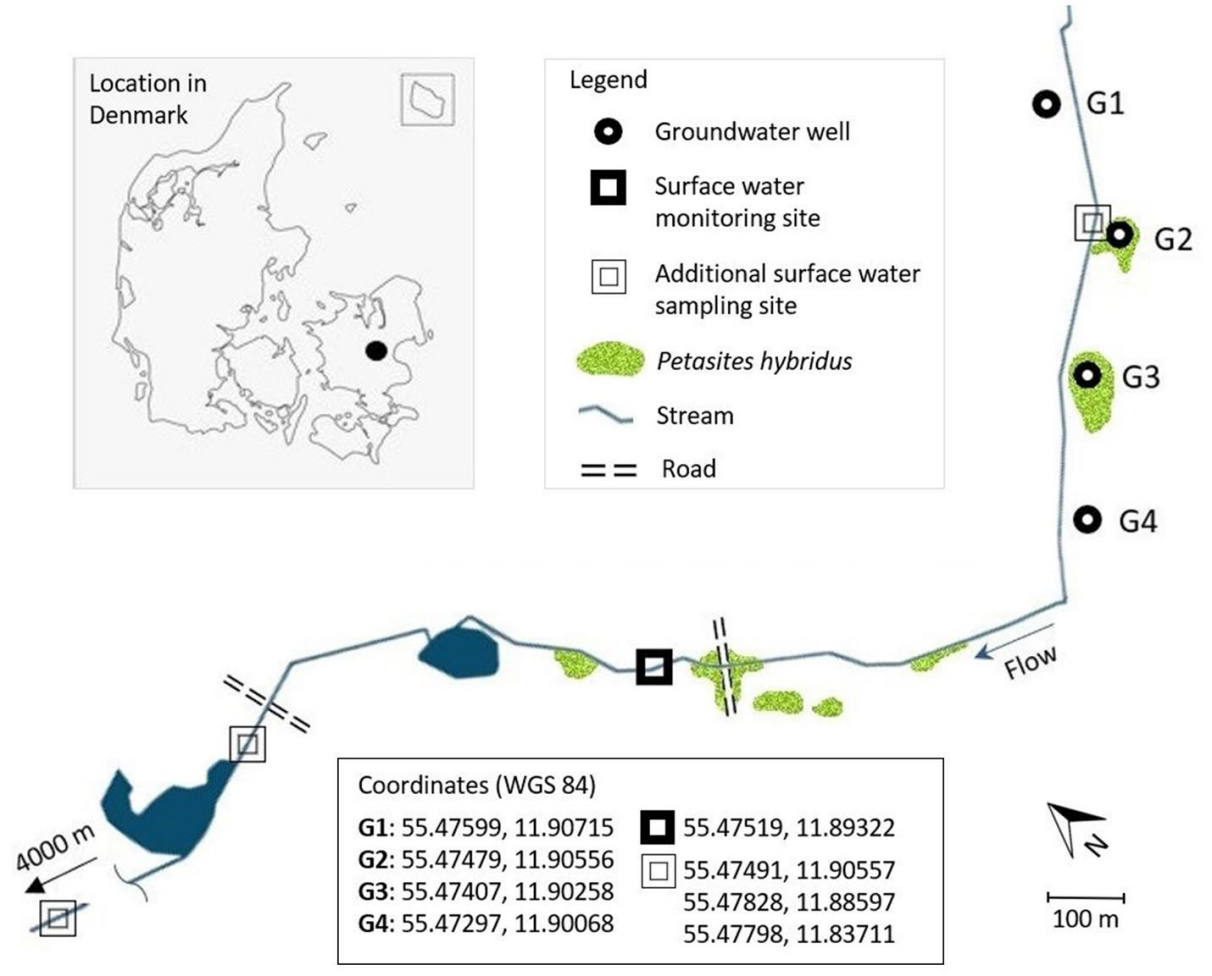
A map of the monitoring site with distribution of butterbur (*Petasites hybridus*).

**Figure 3:**
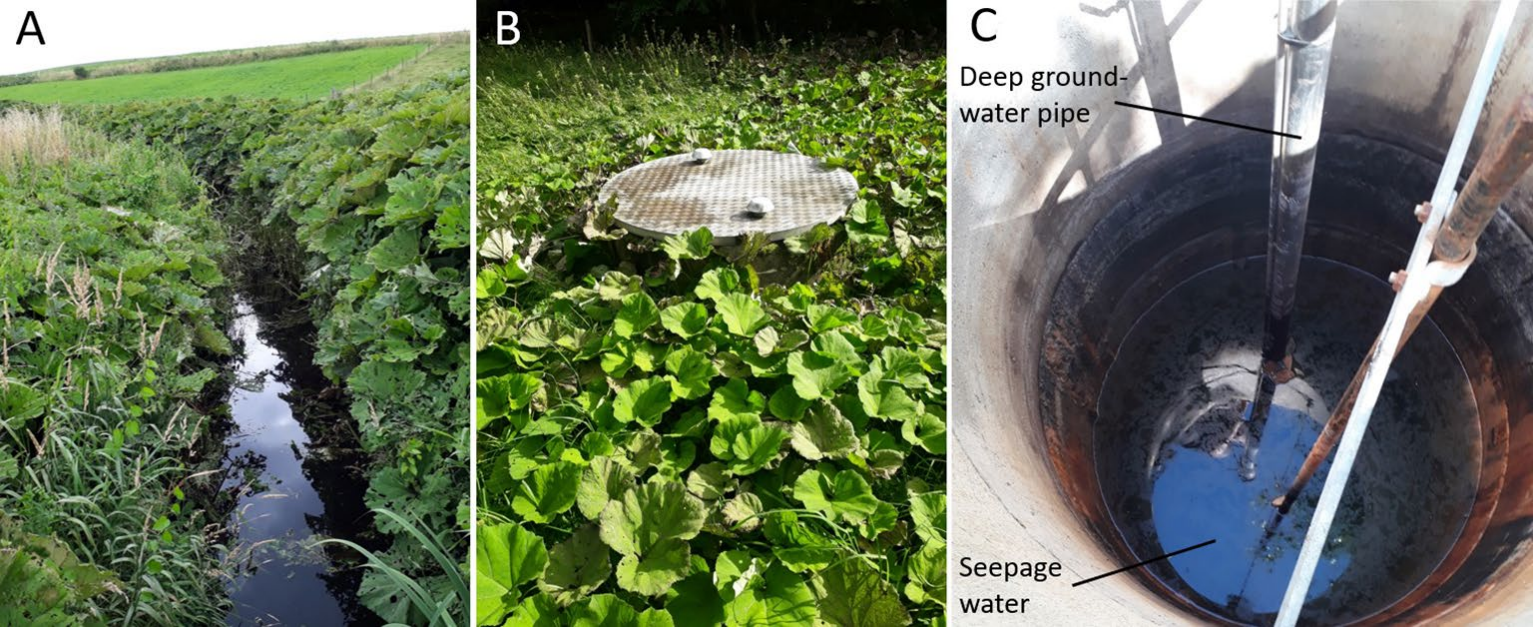
Butterbur on both shores of the sampled stream (**A**) and surrounding the groundwater well G3 (**B**). Open G3 well with seepage water in ~ 2.5 m depth (**C**).

A site with a channelized surface water stream and a system of groundwater abstraction wells was selected in eastern Denmark (Fig. 2). The shores of the stream and the land surface surrounding two of the wells (G2 and G3) were infested with butterbur. Every well consisted of a vertical pipe providing access to deep groundwater (estimated water level of approximately 60 m depth) and a surrounding casing supported by concrete rings that naturally filled with seepage water from the soil (observed water level 2.2–3.0 m depth) (Fig. 3). The waters from different depths are not mixed and the deep groundwater is pumped for municipal water supply.

**Figure 4:**
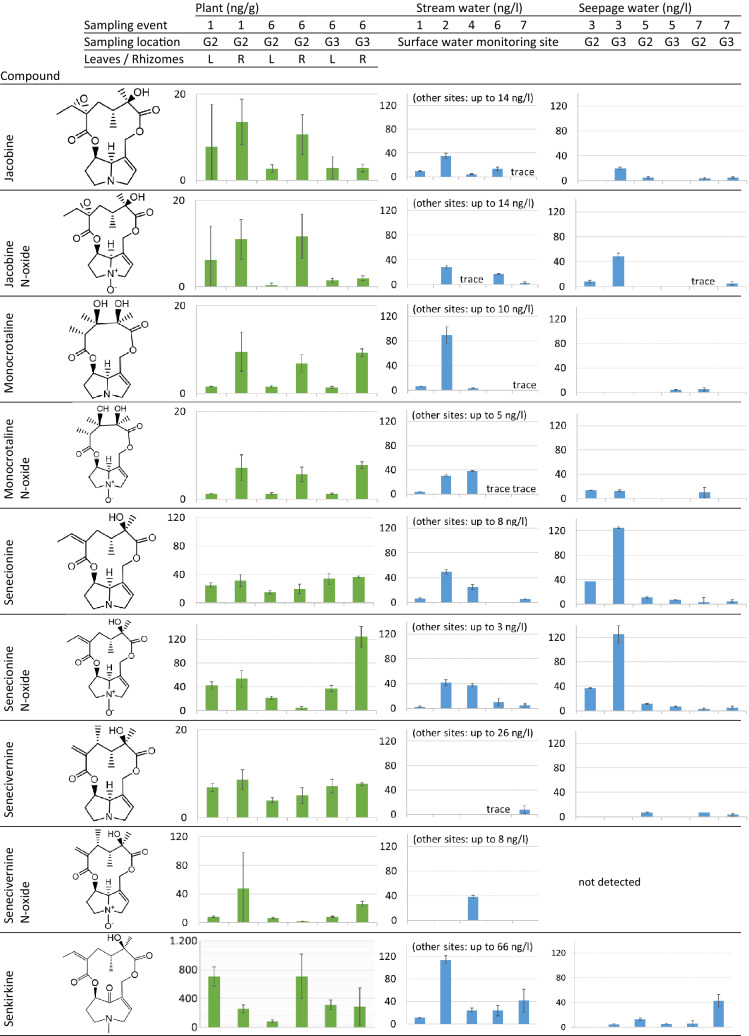
Average concentrations of PAs detected in butterbur plants, stream and seepage waters ± SDs (in alphabetical order). The graphs of PAs in the plants have different scales on the y-axes. The empty values represent no detection. “Other sites” in the stream water column refer to additional surface water sampling sites mapped in Fig. 2. The dates of the sampling events are listed in Table 2 of the SM.

A qualitative test revealed 9 cyclic diester unsaturated PAs in butterbur leaves sprouting near well G2 in April 2019: jacobine, jacobine N-oxide, monocrotaline, monocrotaline N-oxide, senecionine, senecionine N-oxide, senecivernine, senecivernine N-oxide and senkirkine (hereafter referred to as type 1 PAs). Subsequent quantitative analyses of matured butterbur leaves and rhizomes sampled near wells G2 and G3 on the dates listed in Table 2 of the supplementary material (SM) revealed the same PA compositions with concentrations provided in Fig. 4. To the best of our knowledge, monocrotaline and monocrotaline N-oxide have not been reported in *Petasites* sp. before.

A total of 21 PAs were detected in the stream water and in the seepage water of wells G2 and G3. They comprised all the type 1 PAs, together with lower concentrations of 12 unsaturated PAs that were not detected in the butterbur (hereafter referred to as type 2 PAs). Type 2 PAs comprised cyclic diesters (erucifoline, retrorsine, retrorsine N-oxide, seneciphylline), open chain diesters (echimidine, echimidine-N-oxide, lasiocarpine) and monoesters (europine, europine N-oxide, heliotrine, intermedine, intermedine N-oxide). The concentrations of individual type 1 PAs measured in the stream water and in the seepage water in wells G2 and G3 are presented in Fig. 4. No PAs were detected in the deep groundwater, in seepage waters of the control wells G1 and G4, or in blank water sampled as the field controls.

PAs were detected in the stream water and in the seepage water in wells G2 and G3 during all sampling events. In all sampled matrices the concentrations of N-oxides were similar to the concentrations of the equivalent free base compounds. The most frequently found and abundant PAs in the various sampled water corresponded to the most abundant PAs in the plants (senkirkine, senecionine, and senecionine N-oxide). No other PA metabolites were detected, whereas N-oxides are known to be the subject to metabolic back-transformation to corresponding free base PAs^42,53^. In order to be compatible with the recommended thresholds set by health authorities for PAs in orally consumed products^24,57,62^, further risk assessment will be based on the total unsaturated PA concentrations.

**Figure 5:**
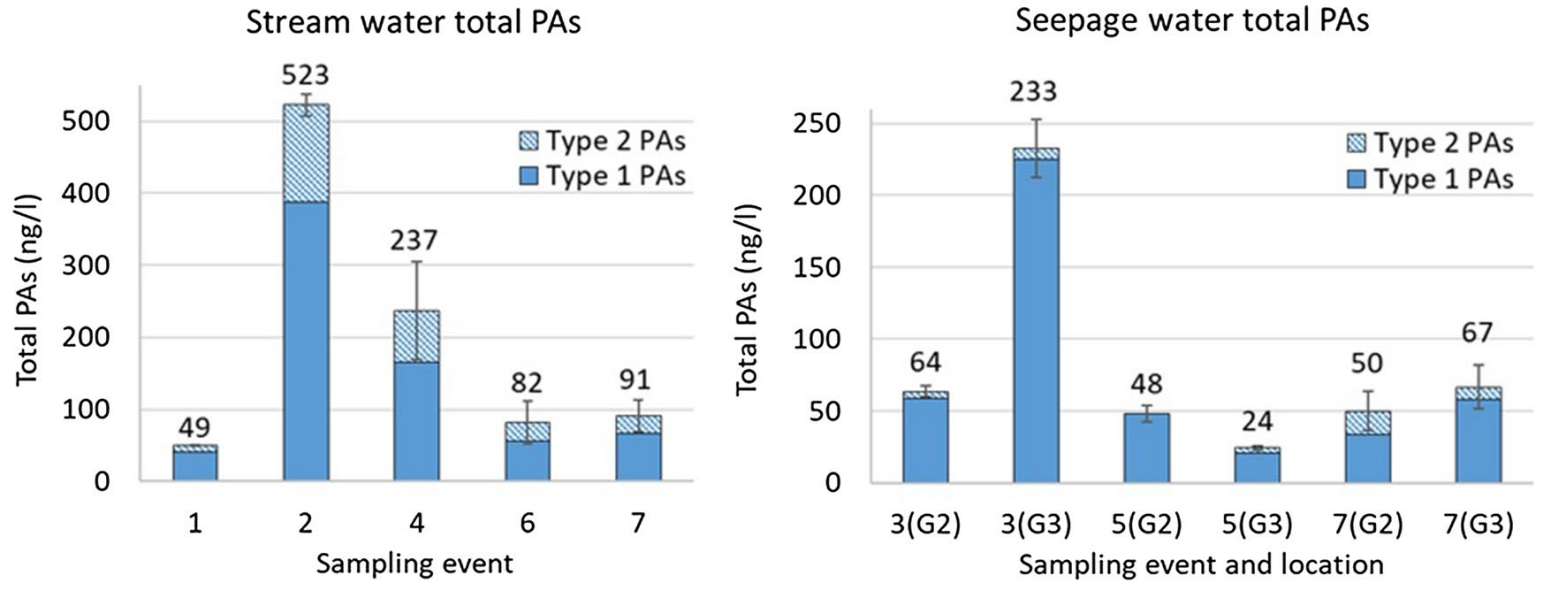
Concentrations of total PAs in the stream at the surface water monitoring site (**A**) and in seepage waters in the wells (**B**) ± SDs of the total PA concentrations in triplicate samples. The type 1 PAs represent the sum of nine PAs that were detected in plants and water (Fig. 4), whereas the type 2 PAs represent the sum of twelve PAs that were detected in water only (Table 1 of the SM). The dates of the sampling events are listed in Table 2 of the SM, the seepage water sampling locations mapped in Fig. 2.

The base stream flow (sampling events No. 1, 6 and 7 (Table 2 of the SM)) had average total PA concentrations in the surface water monitoring site from 49 to 91 ng/L (Fig. 5). Intense rapid rain between sampling events No. 1 and 2 (13 mm recorded by the Danish Meteorological Institute) resulted in a tenfold increase in the total PA concentration, implying simple and quick toxin washoff and release from the plants or the soil. The segmentation of the stream into stagnant water patches due to summer drought conditions (sampling event No. 4) resulted in PA concentrations approximately 3 times higher than at the base flow.

Total PA concentrations in the well seepage waters were highest during sampling event No. 3 (Fig. 5) which corresponded with the largest biomass of adjacent butterbur. The greater plant coverage of the well (well G3, Fig. 2) was associated with higher PA concentrations in the well water. There were fewer plants around the wells at subsequent sampling events due to regular biomass cutting and removal by a maintenance company to ensure access to the wells. This biomass removal possibly caused reduced PA concentrations.

The concentrations of individual type 2 PAs that are presented in Fig. 5 did not exceed 10 ng/L, except in the stream water under non-base flow conditions (108 ng/L of retrorsine and 27 ng/L of seneciphylline in sampling event No. 2, and 56 ng/L of erucifoline and 46 ng/L of retrorsine N-oxide in sampling event No. 4). Type 1 PAs comprised cyclic diesters only, whereas type 2 PAs also included open-chain diesters and monoesters (Table 1 of the SM). Open-chain diester and monoester PAs have not been reported in butterbur in this or in previous studies^4^.

## Discussion

This research reports PAs from butterbur in surface water for the first time, and provides evidence for our starting hypothesis that PAs can naturally leach from butterbur into water and make it unsafe to drink. Several previous studies identified PAs as soil or surface water contaminants^70–72^. Experimentally determined octanol–water partition coefficients infer little potential PA sorption to soil^73^. The surface waters adjacent to PA-producing plants may show relatively high PA concentrations (up to 530 μg/L^70^) and thus contribute to the overall exposure of farm and wildlife animals to PAs. Furthermore, this study illustrates PA leaching from PA-producing plants to groundwater wells via seepage water. Open bottomed shallow groundwater wells are common in remote one-household settlements with no centralized water supply in both developing and developed countries. These wells are frequently subject to pollution of organic matter, nitrate and pesticides that seep from farming areas and noncentralized sanitation. To the best of our knowledge PAs in well water have not been examined before.

Other known PA producing plants were not observed in the studied area and the control wells contained neither PA types. That implies that all PAs in water likely originated from butterbur. The highest concentrations of type 2 PAs in the seepage water were observed in sampling event No. 7 (October) at which time there were the highest proportion of naturally wilted and decaying butterbur. The type 2 PAs may have originated from other unknown sources, transformed in decaying biomass or in the water from the type 1 PAs.

According to the recommended maximum daily intake of 23.7 ng/kg body weight of unsaturated PAs, daily intake of 2 L of well water with a PA concentration of 233 ng/L, or 2 L of surface water with 523 ng/L corresponds to 30% and 60% of the maximum allowable intake for a 70 kg healthy human. The stream and shallow well waters reported in this study caused no harm to human health only because these waters were not utilized for drinking. The analysed exploited deep groundwater contained no PAs because this water was pumped from beneath impermeable geological layers with no contact with leachates from the butterbur. However, the groundwater pipes immersed in contaminated seepage water (Fig. 3C) pose a potential risk to groundwater. Poorly drilled or maintained deep groundwater wells are subject to the downward transmission of water carrying microbiological and hazardous anthropogenic contaminants^74–76^.

Microbiological and anthropogenic chemical pollution is methodically monitored and regulated in drinking water by law. The Directive of the European Parliament and of the Council on the protection of groundwater against pollution and deterioration (2006/118/EC) imposes EU member states to terminate exploitation of water from groundwater wells if active substance of any pesticide, including its relevant metabolites, degradation and reaction products exceeds the 100 ng/L threshold^77^. Threshold violations have been reported in Italy, the Netherlands, Belgium, Sweden, Finland and Denmark, leading to the closure of many drinking water wells^74^.

Plant-produced toxic compounds can locally outcompete anthropogenic chemicals in quantity because of their continuous production and comparatively high concentrations in plants^78^. Invasive and foreign ornamental species tend to contain highly toxic natural compounds. Inedible parts of agricultural crops contain natural toxins that could possibly also be released to larger land areas such as carcinogenic quinolizidine alkaloids in leaves and blossoms of lupin, and the acute toxic steroidal alkaloid solanine in leaves and stems of potato^79–81^. To some extent, the natural toxins are regulated in food. Plant toxins not entering the human food chain are generally regarded as not harmful to humans. However, their aquatic toxicity and waterborne ingestion by humans remain largely unknown. In addition to direct harm, ingested plant toxins can interact with other contaminants in humans creating synergies and cumulative adverse health effects^82^.

Anthropogenic activities directly or indirectly stimulate the spread of many invasive species. Studies show that among toxic invasive plants, plants producing PAs and other alkaloids are the most common^83^. For example, the genus *Cytisus* comprises about 50 species of invasive PA-producing flowering plants. Many other plant-produced toxic alkaloids can be stable in water, like alkaloids entailing toxic persistent heterocyclic triazole rings.

The PA releasing butterbur tends to invade moist and disturbed lands like shaded sides of roads, bridge embarkments, the shores of artificial surface water bodies, edges of ditches, channelized streams and water wells. According to the Global Biodiversity Information Facility, the species is omnipresent in most of western and northern Europe, common in the rest of the Europe and occasionally found across North America^84^. No mitigation operations of these species exist. In northern Europe climate change is leading to an increase in precipitation that together with various land management activities will further stimulate the spread of butterbur, leading to an increased risk of PA contaminated water. The absence of regulations on natural toxins in water has resulted in a lack of suitable methodologies for their monitoring and identification. This study illustrates the need for the advancement of procedures to monitor persistent plant toxins and indicates the need for water cleaning technologies to facilitate their removal.

## Methods

### Sample collection

Stream water was sampled over a 4-month period from maturation to decay of adjacently growing butterbur. To identify any PA washoff effects, stream sampling events occurred during base flow conditions, shortly after intense precipitation and during drought conditions. In addition, groundwater and seepage water inside groundwater wells were sampled from two wells with adjacently growing butterbur and two wells without. The three water types, the leaves and the rhizomes of the plant were analysed. The concentrations of individual and total PAs were examined and compared.

Butterbur plants were sampled by cutting 3 whole average-height leaves from the ground surface and digging 10–15 cm segments of 3 rhizomes. Each sample was collected approximately 5 m from one another. The sampled biomass was frozen within 8 h at – 20 °C prior to analyses. The stream water was sampled at the surface water monitoring site during the dates listed in Table 2 of the SM. Intense precipitation resulted in a sudden flow increase, whereas dry periods from July to September resulted in no water movement or dry stream bed. Supplementary stream water samples were irregularly taken at 3 additional surface water sampling sites (Fig. 2).

The seepage water was sampled by manually scooping it from the top 50 cm in 1 L bottles with an extension stick. Deep groundwater was sampled using a pump. The samples of all water types were taken in triplicates of 1 L in polypropylene bottles and frozen within 8 h at – 20 °C prior to analyses. To avoid any possible cross-contamination, no plant samples were taken during groundwater sampling events (Table 2 of the SM). A PA-free blank water was sampled, processed and analysed among all surface and groundwater samples as a field control.

### Analytical

All plant and water samples were prepared by the method described in Hama and Strobel^70^. In short, three plants from each sampling point were finely chopped into 2 mm pieces, vigorously mixed, 0.1 g was measured into 10 ml amber glass tubes, 10 ml of MeOH was added and the mixture was sonicated for 15 min. Subsequently, the supernatants were transferred to 50 ml centrifuge tubes. Extraction cycles with additional MeOH and sonication from the remaining plant materials were repeated twice, resulting in a total of 30 ml of supernatant per sample. The supernatants were centrifuged for 10 min at 2100 g and dried under gentle nitrogen flow in a heating block at 40 °C. The dried extracts were dissolved in 1 ml 40% (V/V) MeCN and filtered through 0.2 μm polytetrafluoroethylene (PTFE) membrane filters prior to analysis.

The water samples were passed through a 2.5 μm filter paper (Whatman quantitative-Grade 42) under a vacuum and acidified to a pH of 3.0 with 0.1 mol/L formic acid. Solid phase extraction cartridges *Oasis MCX* (6 cc, 150 mg 30 µm particle) were conditioned with 5 ml MeOH followed by 5 ml H_2_O, and then 1.0 L of the acidified water samples were loaded at a flow rate of 10 ml/min. The loaded cartridges were washed with 5 ml 0.065 mmol/L formic acid and successively eluted with 5 ml MeOH and with 10 ml 3:1 (V/V) mixture of A: MeOH and B: 10% ammonia solution. A gentle nitrogen flow dried the eluates in a heating block at 40 °C. The dried extracts were dissolved in 1.0 ml 40% (V/V) MeCN and filtered through a 0.2 μm PTFE membrane prior to analysis.

The PAs were identified and quantified from individual fragmentation patterns with UPLC-MS/MS as described in Hama and Strobel^70^. All PAs were detected in a single chromatographic run with the mass spectrometer set for multiple reaction monitoring (MRM). The ion traces were obtained for apex retention time (t_R_) ± 0.15 min. Each MRM mode recorded only one PA by predefined parent and product ions^85^. In total 30 PAs were monitored based on reported PAs in the literature and from Toxic Plants–Phytotoxins Database^83^ whereas the application of t_R_ would have also recorded other PAs. However, only 21 PAs were detected in the samples. When an analyte was detectable but not quantifiable its concentration was set equal to its limit of detection (LOD)^85^.

The instrumental limits of detection and quantification of different PAs were in the range of 2–7 and 5–9 µg/L, respectively. The solid phase extraction provided a 1000 times concentration of the water samples with a 90% recovery rate^70,85^. Field blanks consisted of laboratory grade deionised water that was brought to the field during each sampling event, sampled, handled, prepared and analysed along with the water samples in order to monitor any possible contamination by the PAs during the sampling and preparation steps. All concentrations of PAs in water blanks were below the limits of detection. The average recovery rate of surrogate (caffeine) was 94% ± 11 (n = 3). The concentrations of all the free base PAs and N-oxides were quantified against certified external standards purchased from Phytolab (Germany).

The concentrations of individual PAs were reported as the average concentrations quantified in triplicate samples. In cases when the individual PAs were quantified in only 2 of the triplicate samples (close to the approximate 5–9 ng/L limit of quantification of the full method), the concentrations of individual PAs were reported as the average of 2 positive samples. If individual PAs were quantified in only 1 of the triplicate samples, the concentrations were not quantified and reported as *trace*. Total PA concentrations (Fig. 5) were reported as the averages of the sums of all PAs quantified in triplicate samples.

## Supplementary information

Supplementary Information 1.
