# Supplementary material for: The invasive butterbur contaminates stream and seepage water in groundwater wells with toxic pyrrolizidine alkaloids
Source: Sci Rep. 2020 Nov 13;10:19784. doi: 10.1038/s41598-020-76586-1 (PMC7666219; doi:10.1038/s41598-020-76586-1)
Supplement: Supplementary file 1 — Supplementary Information 1. [file 41598_2020_76586_MOESM1_ESM.docx]

Supplementary material

The invasive butterbur contaminates stream and seepage water in groundwater wells with toxic pyrrolizidine alkaloids

Vaidotas Kisielius, Jawameer R. Hama, Natasa Skrbic, Hans Christian Bruun Hansen, Bjarne W. Strobel, Lars Holm Rasmussen

Table 1. Average concentrations of type 2 PAs detected in water bodies ± SDs (in alphabetical order). The empty values represent no detection. “Other sites” in the stream water column refer to additional surface water sampling sites mapped in Figure 2.

|  |  |  | Stream water (ng/l) | | | | |  | Seepage water (ng/l) | | | | | |
| --- | --- | --- | --- | --- | --- | --- | --- | --- | --- | --- | --- | --- | --- | --- |
|  | Sampling event |  | 1 | 2 | 4 | 6 | 7 |  | 3 | 3 | 5 | 5 | 7 | 7 |
|  | Sampling location | Surface water monitoring site | | | | | |  | G2 | G3 | G2 | G3 | G2 | G3 |
| Compound |  |  |  |  |  |  |  |  |  |  |  |  |  |  |
| Echimidine | 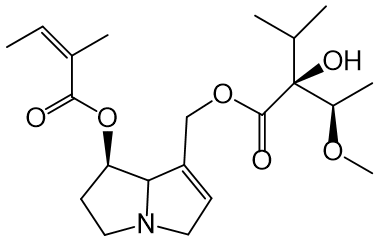 | other sites: up to 4 ng/l | | | | | |  |  |  | trace |  |  |  |
|  |  |  |  |  |  | trace |  |  |  |  |  |  |  |  |
| Echimidine N-oxide | 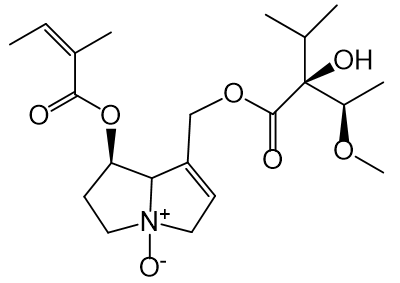 |  | | | | | |  |  |  | 9 ± 9 |  |  |  |
|  |  |  |  |  |  |  |  |  |  |  |  |  |  |  |
| Erucifoline | 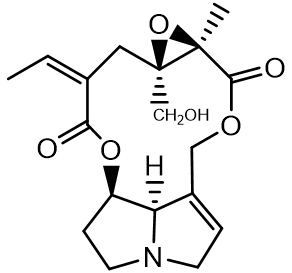 | other sites: up to 13 ng/l | | | | | |  |  |  |  | 4 | 4 ± 1 |  |
|  |  |  |  |  | 56 ±  12 | 3 ± 2 |  |  |  |  |  |  |  |  |
| Europine | 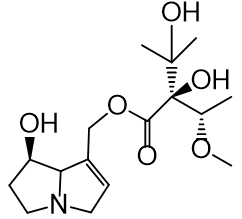 | other sites: trace | | | | | |  |  |  |  |  |  | trace |
|  |  |  |  |  |  |  |  |  |  |  |  |  |  |  |
| Europine N-oxide | 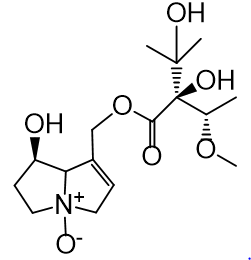 | other sites: trace | | | | | |  |  |  |  |  |  |  |
|  |  |  |  |  |  |  |  |  |  |  |  |  |  |  |
| Heliotrine | 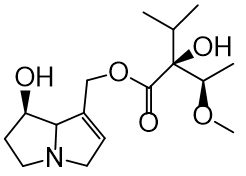 | other sites: up to 10 ng\l | | | | | |  |  |  |  |  | trace | trace |
|  |  |  |  |  |  | 10 ± 5 | 9 ± 7 |  |  |  |  |  |  |  |
| Intermedine | 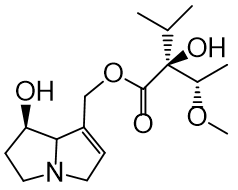 | other sites: trace | | | | | |  |  |  |  |  |  |  |
|  |  |  |  |  |  |  |  |  |  |  |  |  |  |  |
| Intermedine N-oxide | 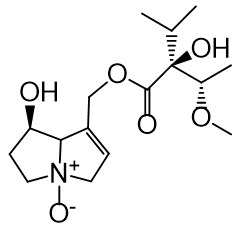 | other sites: up to 14 ng\l | | | | | |  |  |  |  |  |  |  |
|  |  |  |  |  |  |  |  |  |  |  |  |  |  |  |
| Lasiocarpine | 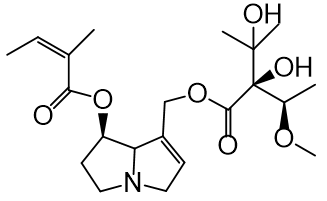 | other sites: up to 24 ng\l | | | | | |  |  |  |  |  | trace | trace |
|  |  |  |  |  |  |  | 9 ± 3 |  |  |  |  |  |  |  |
| Retrorsine | 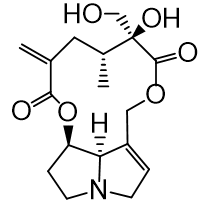 | other sites: up to 9 ng\l | | | | | |  |  |  |  |  | 2 ± 1 |  |
|  |  |  | 9 | 108 ± 13 | 4 ± 2 |  |  |  |  |  |  |  |  |  |
| Retrorsine N-oxide | 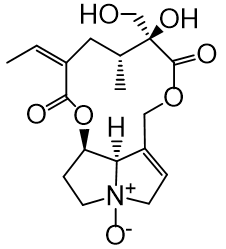 | other sites: trace | | | | | |  |  |  |  |  |  |  |
|  |  |  |  |  | 46 ± 6 |  | trace |  |  |  |  |  |  |  |
| Seneciphylline | 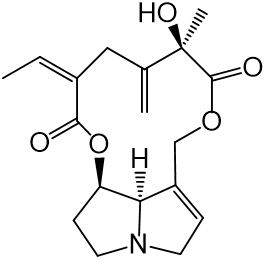 | other sites: up to 9 ng\l | | | | | |  | 5 ± 1 | 8 ± 1 |  |  |  |  |
|  |  |  |  | 27 ± 3 |  | trace | trace |  |  |  |  |  |  |  |

Table 2. The dates of sampling events. The "x" denote samples taken.

| Sampling event | Date (2019) | Plant | Stream water (flow conditions) | Well water |
| --- | --- | --- | --- | --- |
| 1 | June 14 | x | x (base flow) |  |
| 2 | June 16 |  | x (intense flow) |  |
| 3 | July 3 |  | x (no water) | x |
| 4 | August 16 |  | x (no flow) |  |
| 5 | September 12 |  | x (no flow) | x |
| 6 | October 1 | x | x (base flow) |  |
| 7 | October 8 |  | x (base flow) | x |
